# Limited knowledge of diabetes in patients attending an outpatient diabetes clinic at a referral hospital in Zimbabwe: a cross-sectional study

**DOI:** 10.11604/pamj.2018.29.144.12301

**Published:** 2018-03-05

**Authors:** Esther Mufunda, Åsa Ernersson, Katarina Hjelm

**Affiliations:** 1 Department of Health Sciences, Zimbabwe Open University, Harare, Zimbabwe; 2 Department of Social and Welfare Studies, Linköping University, Norrköping, Sweden

**Keywords:** Cross-sectional study, determinants, diabetes awareness, diabetes knowledge, diabetes mellitus, Zimbabwe

## Abstract

**Introduction:**

Diabetes mellitus (DM) has increased globally, with a significant increase noted in African communities. Self-care health-related behavior is determined by beliefs about health and illness which are based on the person?s knowledge of diabetes. The present study aimed to assess patients' diabetes awareness and level of diabetes knowledge in Zimbabwean adults with diabetes attending an outpatient diabetes clinic at a main referral hospital.

**Methods:**

In this cross-sectional descriptive study, the Diabetes Knowledge Test (DKT) was used to measure 96 (71 women and 25 men) patients' knowledge of diabetes and their treatment. Both descriptive and analytic statistical methods were used.

**Results:**

Most respondents had poor knowledge in all the three knowledge categories, total knowledge of diabetes, general knowledge of diabetes and knowledge of insulin use. Major knowledge gaps were noted related to insulin use, glycemic control and diet. Attending DM classes was significantly associated with general knowledge about diabetes (p 0.026) while the level of education was an independent determinant of Total Knowledge and Insulin use knowledge scores.

**Conclusion:**

The identified knowledge gaps need to be addressed to control and minimize diabetes mellitus-related complications.

## Introduction

Diabetes Mellitus (DM) is a multifaceted disease whose devastation breaches all demographics, including that of age, gender and ethnicity. The prevalence of this disease is growing exponentially, having increased over 50% throughout the past thirty years and currently affecting more than 400 million people worldwide, with 46% being undiagnosed [1]. It is anticipated that the increase will be up to 642 million people by 2040 [1] and proportionately greater in developing countries, especially in sub-Saharan Africa, where the number is expected to increase from 19.8 million in 2013 to over 40 million by 2035 [2]. Currently, Zimbabwe ranks fourth amongst the African countries with the highest prevalence rate of diabetes. It is also estimated that a staggering 80% of people with diabetes live in low- and middle-income countries and the socially disadvantaged in any country are the most vulnerable to the disease, with most deaths occurring under the age of 70 years [3], with Type 2 DM being the predominant, accounting for 70-90% of the cases [4]. The disease which is now regarded as a pandemic is rapidly spreading in developing countries and particularly affecting poor populations in sub-Saharan Africa [5, 6]. The prevalence of diabetes in African communities is on the increase, with ageing of the population and lifestyle changes from a traditional healthy and active life to a modern sedentary, stressful life and over-consumption of energy-dense foods [7], associated with rapid urbanization and westernization. Diabetes mellitus is a chronic illness that requires continuing medical care and patient self-management education related to diet, exercise and medication in order to prevent acute and long-term complications. Glycaemic control plays a key role in the outcome of diabetes mellitus and several large-scale trials have demonstrated that comprehensive interventions which include self-management can prevent complications [8]. The management of diabetes mellitus mainly depends on the patients' ability to do self-care in their daily lives. Thus, patient education is always considered an essential element of DM management [9], including collective consensus and public investment in interventions that are affordable, cost-effective and based on the best available science [3]. Studies have concluded that patients with diabetes in some African countries have limited knowledge about the disease, its management and patient self-care [10, 11-13]. As part of self-care, health-related behavior is determined by beliefs about health and illness which are based on the person's knowledge of diabetes [7, 14, 15]. Our previous study of Zimbabweans with diabetes, focusing on beliefs about health and illness, indicated that limited knowledge about the disease affected self-care and health-seeking behavior among Zimbabwean males and females with diabetes [16]. In a recent study that focused on the level and determinants of diabetes knowledge in Zimbabwean adults with diabetes mellitus, we reported that patients with diabetes had major knowledge gaps regarding diet, glycemic control and insulin use [17]. However, the participants were recruited from a patient organization that offered various activities such as patient education in diabetes and opportunities to meet other patients with diabetes to share their experiences. One can assume that patients involved in patient associations have greater knowledge and awareness of their health and self-care behavior than patients in a more general population like the ones who participated in the current study. Participants in that study generally had a higher educational level than the rest of the population in Zimbabwe. Therefore, this study investigated the knowledge of diabetes in a general population of persons diagnosed with diabetes visiting an outpatient diabetes clinic at a main referral hospital to control their disease.

**Aim of the study:** The study aimed to assess patients' diabetes awareness and level of diabetes knowledge in Zimbabwean adults with diabetes attending an outpatient diabetes clinic at a main referral hospital

## Methods

**Study design:** A cross-sectional descriptive study design was used to assess the respondents' diabetes awareness, level and determinants of diabetes knowledge.

**Respondents:** By convenience sampling we recruited 96 adults, 71 females and 25 men who met the inclusion criteria: being diagnosed with diabetes mellitus for ≥ 1 year, mentally sound to give informed consent and being conversant with either English or Shona (the most frequently used languages in the country) [18].

Data collection instrument: A standardized self-report questionnaire including both socio-demographic and diabetes-related background data was used [17]. To measure patients' knowledge of diabetes and their treatment we used the Diabetes Knowledge Test (DKT). DKT is a valid and reliable instrument [19] which was translated into Shona [17]. DKT measures beside general knowledge about diabetes (14 items), patients' knowledge of insulin-use (9 items). The sum of the two subscales forms the total knowledge about diabetes (23 items) [19], for details see Mufunda et al [17].

**Data collection:** Data was collected twice a week over a period of a month during 2013 from patients who came to the Outpatient Diabetes Clinic at a governmental referral hospital in Zimbabwe. The first author, who is a registered nurse, distributed the questionnaires to those who met the inclusion criteria, with the assistance of a trained assistant, who is also a registered nurse. Literate patients filled out the questionnaires themselves while those with writing challenges were assisted by the researcher and the trained research assistant. If needed, the two clarified and answered questions from the respondents during the completion of the questionnaires. A total of 50-60 minutes was allocated to complete the questionnaire.

**Ethical considerations:** Permission to conduct this study was obtained from the Research Ethics Committee of the hospital. The study was performed in accordance with the Helsinki Declaration [20] and written informed consent was obtained from all the respondents.

**Data analysis:** Descriptive statistics were used to describe patients' demographic and medical characteristics and their diabetes knowledge scores. Percentages and frequencies were used for the categorical variables, while means and standard deviations were calculated for the continuous variables. Bivariate correlations were performed between knowledge of insulin use and employment, level of education, treatment regimen and attendance in diabetes class. The chi-square test was employed for categorical variables while student's t-test was used for group comparisons and one-way analysis of variance (ANOVA) was used to compare the differences between the groups (> 2 groups) for normally distributed variables. For the non-normally distributed variables, the non-parametric Mann-Whitney U-test was performed to compare two groups while the Kruskal-Wallis test was used to compare more than two groups. Pearson's Chi-squared test was used to ascertain any association between two qualitative variables. Three multiple logistic regression analyses were performed to identify any independent association between the participants' socio-demographic and diabetes-related characteristics and low knowledge levels (general knowledge, insulin use knowledge and total knowledge). The analysis was performed in steps, with all independent variables included in the first step. Subsequently, step by step, the variable in the previous step with the highest p-value was excluded from the model. As dependent variables the third of the participants with the lowest knowledge level in the respective subscale was chosen [21]. Data was analyzed using SPSS, version 20 (SPSS Inc, IL, USA), with the significance level set at p < 0.05. The total knowledge score was determined by awarding one point for each correct answer and a zero for a wrong answer or no response. The total knowledge score carried a maximum of 23 and was categorized as follows: < 11= poor knowledge, 11-17 = average knowledge and > 17 = good knowledge. The general knowledge score was graded out of a maximum of 14 and categorized as: < 7= poor, 7-11= average, > 11 = good, while the insulin use knowledge carried a maximum of 9 and was categorized as follows: < 5 = poor, 5-7 = average and > 7 = good [22]. To identify the knowledge gaps, all the questions that were with a score of less than 50% were recorded.

## Results

**Respondents' socio-demographic and diabetes-related data:** Out of a total of 108 completed questionnaires, 12 were not included in the analysis because they were incompletely filled in, giving a return rate of 89%. A total of 96 respondents were considered and out of these, 71 were females and 25 were males. The mean age of respondents was 48 (range 17-95) years, and no significant difference were found between men and women. Most of the respondents were married (57.3%) and had low-level education with 14% reporting tertiary/college-level education; while over half of the respondents, 56 (58.3%) were unemployed. A third of the respondents had suffered from DM for 10-15 years and most (53.1%) were on oral agents. The socio-demographic and diabetes-related characteristics of the respondents are summarized in Table 1.

**Table 1:** Socio-demographic and diabetes-related characteristics of the respondents

Variable	Frequency n (%)
**Gender**	
Male	25 (26.0)
Female	71 (74.0)
**Marital status**	
Unmarried	20 (20.8)
Married	55 (57.3)
Divorced/separated	6 (6.2)
Widow/widower	15 (15.6)
**Educational level**	
Primary school	26 (27.1)
Secondary school	47 (49.0)
Tertiary/college	14 (4.6)
University < 2 years	3 (3.1)
University > 3 years	6 (6.2)
**Employment status**	
Unemployed	56 (58.3)
Gainfully employed	32 (33.3)
Sick leave	0 (0)
Retired	8 (8.3)
**Duration of diabetes in years**	
≤ 3	21 (21.9)
4–9	27 (28.1)
10–15	32 (33.3)
≥ 16	16 (16.7)
Diabetes treatment regimen	
Diet only	5 (5.2)
Oral agents	51 (53.1)
Insulin	27 (28.1)
Combination of insulin and oral agents	13 (13.5)
**Diabetes-related complications**	63 (65.6)
**Attendance at diabetes class**	43 (44.8)
**Duration of diabetes class attendance in years**	
≤ 3	30 (31.2)
4–9	19 (19.8)
≥ 10	23 (24.0)
Missing	24 (25.0)

**Level of diabetes knowledge:** Overall respondents' mean score on DKT was 10.4 (2.8) considering total knowledge, for general knowledge 6.4 (1.9) and for insulin use knowledge 4.1 (1.8) (data not shown). No significant difference in diabetes knowledge was found between men and women as shown in Table 2. In general, knowledge was poor in all three categories (total knowledge, general knowledge and insulin use knowledge) (Table 3).

**Table 2:** Comparison between males’ and females’ mean knowledge scores about diabetes

Knowledge category	Males	Females	
	Mean (SD)	Mean (SD)	p-value
Total knowledge (out of 23)	10.6 (2.8)	10.3 (2.8)	0.49
General knowledge (out of 14)	6.6 (2.8)	6.4 (1.9)	0.89
Insulin use knowledge (out of 9)	4.04 (1.5)	3.96 (1.9)	0.83

**Table 3:** Respondents’ knowledge about diabetes, measured with diabetes knowledge test (DKT)

Category	Frequency n (%)	Description of knowledge level
**1. Total knowledge score (out of 23)**		
< 11	51 (53.1)	Poor
11–17	44 (45.8)	Average
≥ 18	1 (1.0)	Good
**2. General knowledge score (out of 14)**		
< 7	49 (51.0)	Poor
7–11	47 (49.0)	Average
≥ 12	0 (0)	Good
**3. Insulin use knowledge score (out of 9)**		
< 5	56 (58.3)	Poor
5–7	39 (40.6)	Average
≥ 8	1 (1.0)	Good

**Diabetes knowledge gaps:** Knowledge deficit, defined as over 50% incorrect answers, was found for 13 questions. In Table 4 the 10 most incorrectly answered questions are shown. The most frequently incorrectly answered question “Which of the following is a “free food”' had 92% incorrect answers and 77% incorrectly answered the statement “The Diabetes diet is”. In addition to these two questions, there were another four items on which more than 70% of the respondents answered incorrectly. These were related to signs of ketoacidosis (89%), how blood glucose is affected by an infection (83%) or by physical activity (74%) and the purpose of testing HbA1c (77%). Comparison of the respondents' socio-demographic and diabetes-related characteristics with their mean diabetes knowledge scores showed that there were no significant differences since most of the mean scores were below the mean scores for each knowledge category. However, the period of attending DM classes was significantly associated with general knowledge about diabetes (p = 0.026) while the respondents' employment status was significantly associated with Insulin use knowledge (p = 0.013), as shown in Table 5, Table 6. The results also showed that attending diabetes classes did not make any differences in the respondents' mean knowledge scores in all three categories (general knowledge, insulin use knowledge and total knowledge).

**Table 4:** The 10 most incorrectly answered questions

Incorrectly answered (%)	
**Q1. Which of the following is a “free food”**	91.7
any unsweetened food	
any dietetic food	
any food that says “sugar free” on the label	
any food that has less than 20 calories per serving*	
**Q2. Signs of ketoacidosis include:**	88.5
shakiness	
sweating	
vomiting*	
low blood glucose	
**Q3. Infection is likely to cause:**	83.3
an increase in blood glucose*	
a decrease in blood glucose	
no change in blood glucose	
**Q4. Glycosylated hemoglobin (or HbA1_c_) is a test that is a measure of your average blood glucose level for the past:**	77.1
day	
week	
6–10 weeks*	
6 months	
**Q5. The diabetes diet is:**	77.1
The way most Zimbabwean people eat	
A healthy diet for most people*	
Too high in carbohydrate for most people	
Too high in protein for most people	
**Q6. For a person in good control, what effect does exercise have on blood glucose?**	74.0
lowers it*	
raises it	
has no effect	
**Q7. If you have taken intermediate acting insulin [NHP or Lente], you are most likely to have an insulin reaction in:**	69.8
1–3 hours	
6–12 hours*	
12–15 hours	
more than 15 hours	
**Q8. Which should not be used to treat low blood glucose?**	68.8
3 hard candies	
½ cup orange juice	
1 cup diet soft drink*	
1 cup skim milk	
**Q9. You realize just before lunch time that you forgot to take your insulin before breakfast. What should you do now?**	63.5
skip lunch to lower your blood glucose	
take the insulin that you usually take at breakfast	
take twice as much as insulin as you usually take at breakfast	
check your blood glucose level to decide how much insulin to take*	
**Q10. Which one of the following is likely to cause an insulin reaction?**	62.5
heavy exercise*	
infection	
overeating	
not taking your insulin	

* Correct answer in italics, based on Fitzgerald et al

**Table 5:** Comparison of mean knowledge scores according to the respondents’ socio-demographic and diabetes-related data

Variable	General knowledge score(out of 14)Mean ±SD	p-value	Insulin use knowledge score(out of 9)Mean ±SD	p-value	Total Knowledge score(out of 23)Mean ±SD	p-value
**Gender**		**0.890**		**0.833**		**0.493**
Males	6.56±2.0		4.04±1.5		10.60±2.8	
Females	6.37±1.7		3.96±1.9		10.32±2.8	
**Marital status**		**0.594**		**0.266**		**0.922**
unmarried	6.46±1.7		3.90±1.6		10.37±2.6	
Married/ cohabitating	6.38±2.1		4.04±2.0		I0.42±3.0	
**Educational level**		**0.454**		**0.128**		**0.104**
Low educational level [Primary & secondary]	6.22±1.9		3.70±1.8		9.92±2.6	
High educational level [College + university]	7.04±2.0		4.87±1.6		11.91±3.0	
**Employment status**		**0.464**		0.013**		**0.141**
Unemployed	6.25±2.0		3.63±1.8		9.88±2.7	
Gainfully employed	6.77±1.8		4.71±1.7		11.48±2.8	
**Diabetes treatment regimen**		**0.758**		**0.404**		**0.437**
Diet	6.80±1.8		4.00±2.1		10.804.00±3.5	
Oral agents	6.41±2.1		3.61±2.0		10.02±2.9	
Insulin only	6.07±1.6		4.56±1.5		10.63±2.6	
Oral agents + insulin	7.00±1.7		4.23±1.3		11.23±2.5	
**Any diabetes-related complications**		**0.274**		**0.532**		**0.635**
Yes	6.12 ±2.0		3.78 ±1.6		9.88 ±2.6	
No	6.57 ±1.9		4.10 ±1.9		10.67±2.9	
**Attending diabetes class**		0.026**		**0.086**		**0.489**
Yes	6.40 ±2.3		4.53±1.5		10.93 ±3.0	
No	6.43 ±1.6		3.53±1.9		9.96 ±2.6	

** p< 0.05

**Table 6:** Summary of independent determinants of diabetes knowledge multiple logistic regression

Characteristicvariable	GeneralKnowledgeScore(out of 14)	Insulin use knowledgeScore(out of 9)	Totalknowledge score(out of 23)
1. Employment status	p=0.464	P = 0.013**	P = 0.141
2. Attending diabetes classes	P = 0.026**	P = 0.086	P = 0.489

** p< 0.05

**Independent associations of knowledge with the socio-demographic variables:** Estimation of the independent associations between poor knowledge and socio-demographic variables and diabetes-related characteristics was done using multiple linear regression tests. The results showed that the respondents' level of education was an independent determinant of total knowledge score (p = 0.041; 95% CI; b = 0.704, OR = 0.51) and Insulin Use Knowledge (p = 0.010, 95% CI; b = 0.513, OR = 0.55).

## Discussion

The risks of developing diabetes-related complications are influenced by the patient's knowledge and management of the disease [23]. Beliefs about health and illness, depending on knowledge of the disease, also affect self-care and health-seeking behavior [17, 24]. Recently, we reported limited knowledge of diabetes in Zimbabwean adults with diabetes mellitus. Major knowledge gaps were found considering diet, glycemic control and insulin use, in a population that can be considered to have a higher level of education than the general population in the country [17]. In this second study, we investigated diabetes knowledge in a more general population of the Zimbabwean population with diabetes, and found a low level of diabetes knowledge independent of educational level. Previous studies conducted in both developed and developing countries have reported that diabetes knowledge was generally poor among persons with diabetes [25-29].

These findings are consistent with findings from the present study conducted in Zimbabwe, a developing country in the sub-Saharan region. Such findings might suggest the need to re-evaluate the current focus and strategies of public diabetes education programs in Zimbabwe as a way to further strengthen knowledge of diabetes among persons with diabetes. Generally, the majority of the respondents in this study demonstrated poor knowledge about diabetes and insulin use, with the majority scoring below the mean for each knowledge category (mean DKT score 10.40 ± 2.8). In this study, no gender differences were observed although a few previous studies have reported that gender was a determinant of knowledge of diabetes [29, 30] while others reported women to have lower levels of knowledge about diabetes than men [31, 32]. Another study reported women to be more active in self-care and information-seeking than males [16]. This apparent knowledge deficit should be a cause for concern to the health-care providers since patients with low diabetes knowledge levels have been reported to be least likely to comply with diabetes management and instructions from health care professionals [33].

However, findings of this study showed that respondents who attended diabetes teaching sessions reported the same level of understanding as those who had not attended any diabetes classes. This finding could also show that information-giving and teaching sessions scheduled on review days might not be the best approach to diabetes education and care, but patients should be given enough time for teaching and opportunities to demonstrate their understanding through experience sharing, demonstrations, peer teaching, decision-making, problem-solving and planning. Results of a previous study from a National Diabetes Centre [17] showed knowledge gaps related to three areas: glycemic control, insulin use and diet. The results of the present study, with respondents from an outpatient diabetes clinic at a major referral hospital, showed knowledge gaps in thirteen questions related to the same areas. This might indicate that, in a more general population where almost 80% have a low level of education (primary and secondary education), the level of knowledge about diabetes was considerably lower than shown in a similar study [17].

This finding might indirectly point to the general adequacy of nurses' knowledge about diabetes, the quality of the health education given to patients by the nurses and doctors in the diabetes clinics or the level of patients' understanding of the information given. When information is not understood, it seems unlikely that this information is stored in one's memory to be remembered and used at a later time [34]. Diabetes mellitus is a chronic illness that requires continuous medical care and patient self-management education to prevent acute and long term complications [35], an increasing prevalence worldwide and high treatment costs for patients with diabetes. Thus, people with diabetes should receive education about the disease, its prevention and related complications. The absence of properly trained diabetes nurses in outpatient diabetes clinics at major public hospitals and rural health centers in Zimbabwe needs to be looked at seriously. It has been reported that the quality of care and education provided depends on the provider's knowledge and experience [36]. Overall, results from previous studies conducted in both developed and developing countries indicated widespread serious and sustained deficiencies in nurses'knowledge of diabetes and diabetes care [37]. Therefore, public hospitals should aim to give structured educational programs which help people with diabetes to be more empowered to engage in self-care and lifestyle changes. Duran et al [38], further reported that structured educational programs for glucose self-control/monitoring could help to set and achieve goals for nutrition and physical activity. Being gainfully employed was associated with higher knowledge of insulin use (p = 0.013), while attending DM classes was significantly associated with general knowledge about diabetes (p = 0.026). Financial challenges due to unemployment could also affect the patients' compliance with attending reviews at the clinic where diabetes classes were held.

This study has shown an association between attending DM classes and General Knowledge about diabetes. To minimize risk of non-compliance with review dates, there is a need for the nurses working in diabetes clinics to assess the socio-economic status of patients and make referrals to the social workers for travel assistance. Although the quality and content of health information given during DM classes does affect the patients' level of knowledge, adoption of a multidisciplinary approach is needed to address primary and secondary diabetes healthcare issues. The multiple logistic regression tests showed that the respondents' level of education was an independent determinant of the total knowledge and insulin use knowledge scores; this could possibly explain some percentage of the respondents' correct answers. It is easy for people who are more educated to grasp and understand information about their disease and might use this information positively to manage the disease. However, since a few respondents in this study (27%) had only primary-level education, there is need for the nurses to identify patients with low educational levels and customize diabetes educational information to enhance understanding during the limited contact time they spend with patients in the clinics.

**Strength and weakness:** An important strength of this study lies in the use of expert consensus on the appropriate grading criteria of diabetes knowledge scores assessed using a validated standardized DKT questionnaire [19]. A weakness of the study is that it was conducted only in the central region, thus limiting generalizability to other parts of the country. Nevertheless, patients who come to the outpatient diabetes clinic where this study was conducted were referred from all ten regions of Zimbabwe and would therefore be considered to represent the majority of Zimbabweans with diabetes.

## Conclusion

The study revealed areas that need improvement in patients' knowledge of their disease and its management. Identified knowledge gaps need to be addressed by increasing the knowledge among diabetes patients and adapting interventions that are suitable for patients of different competency, gender and cultural backgrounds. This might help support patients in the management of their diabetes and minimize diabetes-related complications. Furthermore, health care providers should be well trained and empowered to develop innovative tools and educational models that enhance the delivery of appropriate health education to patients with diabetes.

### What is known about this topic

- Self-care health-related behavior is determined by beliefs about health and illness which are based on the person's knowledge of diabetes;
- Zimbabwean adults with diabetes mellitus have knowledge gaps regarding diet, glycemic control and insulin use.

### What this study adds

- The low level of diabetes knowledge of the Zimbabwean population with diabetes is independent of educational level;
- Identified knowledge gaps need to be addressed by increasing the knowledge among diabetes patients and adapting interventions that are suitable for patients of different competency, gender and cultural backgrounds.

## Competing interests

The authors declare no competing interests.
